# Prescription of analgesics in patients with rheumatic diseases in Germany: A claims data analysis

**DOI:** 10.1007/s00393-021-00971-y

**Published:** 2021-04-07

**Authors:** K. Albrecht, U. Marschall, J. Callhoff

**Affiliations:** 1 Programme Area Epidemiology and Health Services Research, German Rheumatism Research Centre Berlin, Charitéplatz 1, 10117 Berlin, Germany; 2 Department of Medicine and Health Services Research, Barmer, Wuppertal, Germany

**Keywords:** Entzündlich rheumatische Erkrankungen, Versorgung, Analgetika, Verordnungshäufigkeit, Opioide, Inflammatory rheumatic diseases, Health care, Analgesics, Prescription frequency, Opioids

## Abstract

**Objective:**

To investigate the prescription frequency of analgesics in persons diagnosed with rheumatoid arthritis (RA), axial spondylarthritis (axSpA), psoriatic arthritis (PsA) and systemic lupus erythematosus (SLE) in 2019 using claims data.

**Methods:**

Persons ≥ 18 years insured in 2019 with a diagnosis of RA (M05, M06), axSpA (M45), PsA (M07.0-3) or SLE (M32.1,8,9) were included. Analgesics were identified by the anatomic therapeutic classification (ATC) system. Reported is the percentage of individuals with ≥ 1 analgesics prescription for the respective rheumatic diagnosis in 2019 and for opioids age-standardized in each of the years 2005–2019. In addition, the proportion of long-term opioid use (prescriptions in ≥ 3 consecutive quarter years) in 2006 and 2019 is compared.

**Results:**

Metamizole (29–33%) was the most commonly prescribed analgesic. Nonsteroidal anti-inflammatory drugs (NSAID)/coxibs were prescribed from 35% (SLE) to 50% (axSpA). Of the patients 11–13% were prescribed weak and 6–8% strong opioids. From 2005 to 2019, the proportion of persons with an opioid prescription remained stable, with similar or slightly decreasing proportions of weak opioids and more frequent prescriptions of strong opioids. The proportion of long-term opioid prescriptions increased from 2006 to 2019 from 8.9% to 11.0% (RA), from 6.9% to 9.1% (axSPA), from 7.8% to 9.5% (PsA), and from 7.5% to 8.8% (SLE), corresponding to a 17–24% increase.

**Conclusion:**

The prescription of opioids for persons with inflammatory rheumatic diagnoses is not as high in Germany as in other countries; however, the proportion of long-term prescriptions has considerably increased. The frequent prescription of metamizole is conspicuous.

## Abstract (de)

**Zielsetzung:**

Mithilfe von Abrechnungsdaten wird die Verordnungshäufigkeit von Analgetika bei Patienten mit Diagnose einer rheumatoiden Arthritis (RA), axialen Spondyloarthritis (axSpA), Psoriasisarthritis (PsA) bzw. eines systemischen Lupus erythematodes (SLE) im Jahr 2019 untersucht.

**Methodik:**

Eingeschlossen wurden Versicherte der Barmer ≥ 18 Jahre im Jahr 2019 mit Diagnose einer RA (M05, M06), axSpA (M45), PsA (M07.0–3) oder eines SLE (M32.1, 8, 9). Analgetika wurden über die ATC(Anatomisch-Therapeutisch-Chemisches Klassifikationssystem)-Codes identifiziert. Berichtet wird der Anteil an Personen mit ≥ 1 Analgetikaverordnung für die jeweiligen Krankheitsbilder im Jahr 2019 sowie für Opioide altersstandardisiert in jedem der Jahre 2005 bis 2019. Zusätzlich wird der Anteil an Opioidlangzeitverordnungen (Rezepte in ≥ 3 aufeinanderfolgenden Quartalen) im Jahr 2006 und 2019 verglichen.

**Ergebnisse:**

Metamizol (29–33 %) war das am häufigsten verordnete Analgetikum. NSAR(nichtsteroidale Antirheumatika)/Coxibe wurden zu 35 % (SLE) bis 50 % (axSpA) verordnet; 11–13 % der Versicherten wurden schwach wirksame und 6–8 % stark wirksame Opioide verordnet. Von 2005 bis 2019 blieb der Anteil an Personen mit ≥ 1 Opioidverordnung stabil bei gleichbleibend bzw. leicht rückläufiger Verordnung schwach wirksamer Opioide und häufigerer Verordnung stark wirksamer Opioide. Der Anteil an Langzeitopioidverordnungen stieg von 2006 bis 2019 von 8,9 % auf 11,0 % (RA), von 6,9 % auf 9,1 % (axSpA), von 7,8 % auf 9,5 % (PsA) und von 7,5 % auf 8,8 % (SLE), entsprechend einer Zunahme um 17–24 %.

**Schlussfolgerung:**

Die Verordnung von Opioiden bei Versicherten mit entzündlich rheumatischen Diagnosen ist in Deutschland nicht so hoch wie in anderen Ländern, allerdings ist der Anteil an Langzeitverordnungen deutlich angestiegen. Auffällig ist die häufige Verordnung von Metamizol.

Pain is a major symptom of musculoskeletal diseases that affects almost all patients with inflammatory rheumatic diseases, both in the early phase of the disease and in its further course. In the German National Database of the Collaborative Arthritis Centres (NDB), about half of all patients with rheumatoid arthritis (RA) report moderate to severe pain. With an average duration of the disease of 12 years, this includes a large number of patients with a long-term course of the disease [1]. In the PROCLAIR (Patient-Reported Outcomes with Claims data for health services research in Rheumatology) study, even more than half of the responders with RA, insured by BARMER reported moderate to severe pain [2]. Even though the proportion of patients with severe pain in the NDB is declining, about 15–18% of patients with RA, axial spondyloarthritis (axSpA) or psoriatic arthritis (PsA) and 10% of patients with systemic lupus erythematosus (SLE) report severe pain. Pain caused by rheumatic diseases needs to be considered in a biopsychosocial context and can be addressed at different levels by drug and nondrug interventions. Analgesic drug therapy remains an important component in the treatment of patients with rheumatic diseases [3, 4]. In addition to nonsteroidal anti-inflammatory drugs (NSAIDs) and nonopioid analgesics, opioids are also used as a last option to reduce rheumatic pain. Despite the availability of an increasing number of effective disease-modifying antirheumatic drugs (DMARDs), the prescription of opioids in patients with musculoskeletal conditions, including rheumatologic conditions, has increased in many countries [5]. Whether this also applies to rheumatologic patients in Germany was investigated in this study.

Many rheumatology patients have other comorbidities that are also associated with pain and analgesic use [6]. Analgesics are not only prescribed by rheumatologists, but also by general practitioners and other specialists; in addition, patients purchase over-the-counter drugs from the pharmacy. Due to the wide range of analgesics and indications for them, and due to interdisciplinary prescriptions, we report on the current prescription prevalence of analgesics in insured persons with a diagnosis of RA, axSpA, PsA or SLE using nationwide health insurance data. In these data, all outpatient prescriptions filled at a pharmacy are recorded, regardless of the indication or the prescribing specialist group. The results are compared to data from the NDB and to published data from other countries.

## Methods

BARMER is one of the largest statutory health insurance companies in Germany with around 9 million insured persons nationwide. Outpatient claims data were used. Inclusion criteria were persons ≥18 years, insured in 2019, with ICD-10 diagnosis of one of the following inflammatory rheumatic diseases: RA (M05, M06), axSpA (M45), PsA (M07.0–3) or SLE (M32.1, 8, 9). The diagnosis had to be present in at least two different quarters in 2019.

Analgesics were identified via the Anatomical Therapeutic Chemical (ATC) classification system and were categorised according to the three WHO steps [7]. The following agents were included in the analysis: acemetacin, acetylsalicylic acid (ASA), buprenorphine, celecoxib, diclofenac, fentanyl, etoricoxib, hydromorphone, ibuprofen, indometacin, metamizole, morphine, naproxen, oxycodone, paracetamol, piroxicam, tilidine + naloxone, and tramadol. Of acetylsalicylic acid (ASA) and paracetamol, only the proportion of persons for whom a prescription was issued is included, as these do not require a prescription. Likewise, doses of ibuprofen up to 400 mg are not included.

In order to approximate the severity of the rheumatic disease, it was determined whether the insured persons were prescribed glucocorticoids, conventionally synthetic (cs)DMARDs, targeted synthetic (ts), biologic (b) DMARDs or phosphodiesterase (PDE) inhibitors. The following substances or drug groups were considered: abatacept, apremilast, azathioprine, baricitinib, belimumab, ciclosporin, cyclophosphamide, hydroxychloroquine, interleukin inhibitors, leflunomide, methotrexate, mycophenolate, ocrelizumab, rituximab, sulfasalazine, tumour necrosis factor (TNF-)α inhibitors and tofacitinib.

Selected concomitant diagnoses commonly associated with analgesic prescription were analysed: osteoarthritis (ICD-10 M15–M17), osteoporosis (M80–82), fibromyalgia (M79.70) and malignant neoplasms (C00–C97).

The proportion of persons with at least one prescription for the respective painkiller in 2019 was evaluated. The frequency of prescriptions is reported by gender and age group (18–30, 31–50, 51–70, > 70 years).

To examine trends in the prescription prevalence of opioids, the proportion of persons with at least one prescription of an opioid for the respective diagnosis was determined in each of the years 2005–2019, stratified according to weak (WHO step II: tilidine, tilidine + naloxone, tramadol) and strong opioids (WHO step III: oxycodone, oxycodone + naloxone, morphine, hydromorphone, fentanyl, buprenorphine). For this evaluation, the sample was standardised by age with the European standard population of 2013 [8], so that the age structure is comparable in all years. This ensures that observed effects are not due to differences in the age of persons insured in BARMER over the years.

In addition, it was evaluated how many insured persons were prescribed long-term opioids and metamizole, corresponding to a prescription in ≥ 3 consecutive quarters [9]. For this purpose, the last two quarters of the previous year were also included. The frequency of long-term prescriptions was compared for the years 2006 and 2019.

The lifetime physician numbers (general practitioners 01–03, orthopaedists 10–12, internal rheumatologists 31) were used to identify whether opioids were prescribed by a rheumatologist, orthopaedist, a the general practitioner or another specialist.

Data from the year 2018 from the NDB are reported as comparative data. For this purpose, all patients with a diagnosis of RA, axSpA, PsA or SLE and information on analgesic use were included. The participating rheumatologists documented whether the patients are treated with NSAIDs or coxibs, other analgesics and opioids in the last 12 months.

## Results

Data from *n* = 150,394 (RA), *n* = 30,363 (axSpA), *n* = 19,524 (PsA) and *n* = 5642 (SLE) insured persons, for whom the respective diagnosis was coded in 2019, were included. The diagnosis had to be present in at least two different quarters. The persons had a mean age between 59 (SLE) and 69 years (RA) and 50 (axSpA) to 89% (SLE) were female. Glucocorticoids and csDMARDs were prescribed most frequently for seropositive RA and SLE (including hydroxychloroquine), b/tsDMARDs most frequently for seropositive RA and PsA (including apremilast).

Osteoarthritis was coded in 25% (SLE) to 43% (RA), osteoporosis in 14% (axSpA) to 30% (seropositive RA), malignant neoplasms in 8% (PsA) to 11% (RA) and fibromyalgia in 5% (axSpA) to 7% (PsA, SLE) (Table 1).

**Table 1:** Characteristics of insured persons

	RA			axSpA	PsA	SLE
	All	M05	M06			
*N*	150,394	36,730	113,664	30,363	19,524	5642
Age in years, mean	68.8	67.5	69.2	61.2	61.9	58.7
Female (%)	77.5	79.9	76.7	49.5	66.1	88.9
*Anti-inflammatory medication (%)*						
Glucocorticoids	41.6	55.2	37.2	18.6	32.1	48.7
csDMARDs	35.5	58.0	28.2	10.7	42.8	58.5
b/tsDMARDs/PDE inhibitors	10.2	19.8	7.1	15.3	25.3	5.4
*Concomitant diagnoses (%)*						
Osteoarthritis	42.9	42.2	43.2	28.7	37.2	24.9
Osteoporosis	24.7	29.7	23.1	13.5	15.3	22.9
Malignant neoplasms	10.6	9.6	10.9	9.1	7.7	8.5
Fibromyalgia	5.6	5.1	5.8	4.8	7.1	7.1

*RA* Rheumatoid Arthritis, *M05* seropositive RA, *M06* other/seronegative RA, *axSpA* axial spondyloarthritis, *PsA* Psoriatic Arthritis, *SLE* systemic Lupus erythematosus, *DMARDs* disease-modifying antirheumatic drugs, *b* biologic, *cs* conventional synthetic, *ts* targeted synthetic

csDMARDs: azathioprine, ciclosporin, cyclophosphamide, hydroxychloroquine, leflunomide, methotrexate, mycophenolate, sulfasalazine

b/tsDMARDs/PDE inhibitors: abatacept, apremilast, baricitinib, belimumab, interleukin inhibitors, ocrelizumab, rituximab, TNFα inhibitors, tofacitinib

### Prescription of analgesics

WHO step I painkillers were prescribed to 52% (SLE), 61% (RA) and 62% (axSpA, PsA) of the insured persons. Metamizole was the most frequently prescribed step I analgesic: every third person diagnosed with RA and 30% of the insured persons diagnosed with SLE received metamizole (Table 2). A total of 9% (RA), 6% (axSpA) and 7% (PsA, SLE) had a long-term prescription of metamizole. NSAIDs or coxibs were prescribed from 35% (SLE) to 50% (axSpA), with ibuprofen followed by diclofenac being the most frequently prescribed drugs (Table 2).

**Table 2:** Proportion of insured persons (%) with at least one analgesic prescription of the corresponding substances in 2019

	RA	axSpA	PsA	SLE
Metamizole (N02BB02)	33.1	27.1	29.1	30.1
Ibuprofen^a^ (M01AE01)	26.1	25.7	28.0	22.4
Diclofenac (M01AB05)	11.3	12.4	11.2	6.9
Tilidine+ naloxone (N02AX51)	9.2	7.6	8.0	7.5
Etoricoxib (M01AH05)	8.2	11.3	10.6	5.5
Tramadol (N02AX02)	4.3	4.1	4.2	4.2
Celecoxib (M01AH01)	3.7	5.4	5.3	2.7
Naproxen (M01AE02)	2.6	3.1	3.3	2.0
Oxycodone (+ naloxone) (N02AA05, N02AA55)	3.3	2.6	2.7	2.5
Fentanyl (N02AB03)	2.0	1.2	1.0	1.5
Buprenorphine (N02AE01)	1.1	0.8	0.9	0.7
Morphine (N02AA01)	1.0	0.9	0.9	0.9
Acemetacin (M01AB11)	0.7	1.0	0.7	0.4
Indometacin (M01AB01)	0.4	1.5	0.4	0.2
Piroxicam (M01AC01)	0.4	0.8	0.4	0.2
Paracetamol^b^ (N02BE01)	0.4	0.3	0.3	0.4
Acetylsalicylic acid^b^ (N02BA01)	0.1	0.0	0.0	0.1

The ATC (Anatomical Therapeutic Chemical Classification System) codes of the substances are given in parentheses

*RA* rheumatoid arthritis, *axSpA* axial spondyloarthritis, *PsA* psoriatic arthritis, *SLE* systemic lupus erythematosus

^a^for ibuprofen, only the prescription portion is included (>400 mg)

^b^for paracetamol and acetylsalicylic acid, only the proportion of insured persons to whom a prescription was issued is included

**Fig. 1:**
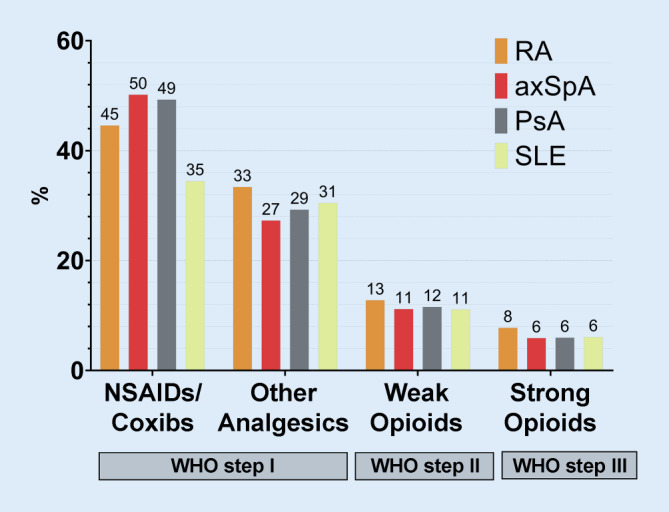
Proportion of insured persons with prescriptions for analgesics according to the WHO (World Health Organization) scheme. *RA* rheumatoid arthritis, *axSpA* axial spondyloarthritis, *PsA* psoriatic arthritis, *SLE* systemic lupus erythematosus, *NSAIDs* nonsteroidal anti-inflammatory drugs

Opioids were prescribed to 17% (SLE, axSpA), 18% (PsA) and 21% (RA) of the individuals, of which 11–13% were weakly effective and 6–8% strongly effective opioids (Fig. 1). Among the step II opioids, tilidine/naloxone was prescribed most frequently, and oxycodone was the most frequently used strong opioid. A total of 74.6% of opioids were prescribed by general practitioners, 3.2% by rheumatologists, 5.8% by orthopaedists and 16.5% by other specialists.

Stratified by age groups, the prescription of NSAIDs decreased in persons aged above 70 years, while metamizole and opioids were prescribed more frequently in the > 70 years age group. Women received analgesics from all WHO steps slightly more often than men (Table 3).

**Table 3:** Prescription frequency of analgesics (%) by age group, including insured persons with RA, axSpA, PsA and SLE diagnosis

	NSAIDs	Other analgesics	Weak opioids	Strong opioids
18–30 years	47.6	19.4	4.4	0.9
31–50 years	51.2	22.8	7.7	2.7
51–70 years	50.3	27.1	10.6	5.1
>70 years	38.4	39.2	15.1	10.6
Women	45.7	34.0	12.9	7.9
Men	43.3	26.3	10.4	5.1

NSAIDs (nonsteroidal anti-inflammatory drugs): ibuprofen, diclofenac, celecoxib, etoricoxib, naproxen, piroxicam, indometacin, acemetacin

Other analgesics: metamizole, acetylsalicylic acid, paracetamol (only prescriptions, over-the-counter is not included in the data)

Weak opioids: tilidine, tilidine + Naloxone, tramadol

Strong opioids: oxycodone (+ naloxone), morphine, hydromorphone, fentanyl, buprenorphine

*RA* rheumatoid arthritis, *axSpA* axial spondyloarthritis, *PsA* psoriatic arthritis, *SLE* systemic lupus erythematosus

No relevant differences were seen in the prescription of analgesics (NSAIDs 47% vs. 44%, other analgesics 33% vs. 34%, opioids 20% vs. 21%) for the RA ICD diagnosis codes M05 (seropositive) and M06 (seronegative/other polyarthritis).

### Prescription of opioids 2005–2019

**Fig. 2:**
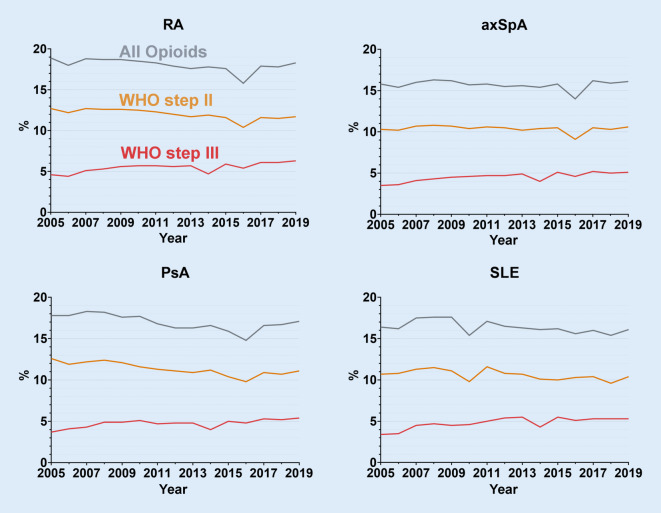
Proportion of insured persons with at least one opioid prescription in the years 2005–2019 (BARMER data, age-standardised). *RA* rheumatoid arthritis, *axSpA* axial spondyloarthritis, *PsA* psoriatic arthritis, *SLE* systemic lupus erythematosus

From 2005–2019, the proportion of persons who were prescribed opioids at least once a year remained relatively constant. The proportion of strong opioids increased by 2–3% for all rheumatic diagnoses, while the proportion of persons receiving weak opioids remained the same or slightly decreased (Fig. 2). The slightly more frequent prescription of opioids among women compared to men remained stable over time.

### Long-term prescription of opioids

The proportion of long-term opioid prescriptions, corresponding to prescriptions in at least three consecutive quarters, increased from 2006–2019 from 8.9% to 11.0% (RA), from 6.9% to 9.1% (axSPA), from 7.8% to 9.5% (PsA) and from 7.5% to 8.8% (SLE). This corresponds to an increase in long-term opioid prescriptions of 24% (RA), 32% (axSpA), 22% (PsA) and 17% (SLE).

### Comparative data from patients documented in the national database in 2018

**Fig. 3:**
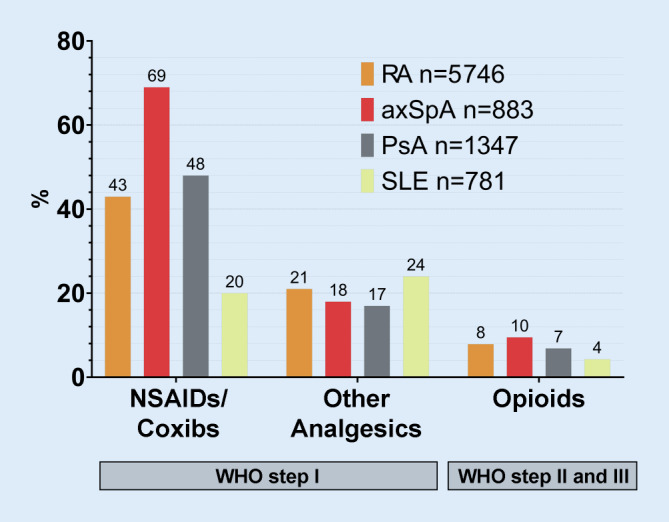
Comparative data from the National database in 2018, including patients with RA, axSpA, PsA and SLE in rheumatology care. *RA* rheumatoid arthritis, *axSpA* axial spondyloarthritis, *PsA* psoriatic arthritis, *SLE* systemic lupus erythematosus

The use of analgesics in patients with established RA as it was documented in the NDB is shown in Fig. 3. The proportion of patients with opioid therapy ranges from 4–10%, depending on the rheumatic diagnosis.

## Discussion

Current data on the prescription of analgesics among insured persons with a diagnosis of RA, axSpA, PsA or SLE show a prescription frequency for analgesics of over 60%, indicating the relevance of analgesic drug prescriptions for rheumatologic patients.

The gender distribution of the insured persons examined for RA and SLE corresponds to the expectations from the NDB; for axSpA and PsA, there are more women among the insured persons. The mean age is 5–10 years above the age average of the NDB for all of the examined rheumatic diagnoses. It needs to be taken into account that the BARMER has a higher proportion of women and a somewhat older population compared to the average of the statutorily insured population. However, comparative evaluations with data from the German Federal Statistical Office for different indications showed only minor deviations. Another possible explanation is that the NDB shows a selection of more severe forms of the diseases due to the participation of specialised arthritis centres, which are often associated with an earlier age of onset, whereas the insurance data contain all cases, including very mild forms of the disease. Glucocorticoid and DMARD prescriptions show expected differences in the four rheumatic diagnoses and confirm the low DMARD prescription in seronegative RA. As the M06 diagnosis is disproportionately frequent and partly nonspecific coding, M05 and M06 were considered separately for RA.

NSAIDs were the most commonly prescribed analgesic for all four diagnoses

NSAIDs were the most commonly prescribed analgesic for all four diagnoses. As first-line therapy for axSpA and also for symptomatic analgesic therapy in the other diagnoses, this corresponds to a guideline-compliant approach [3, 4]. Ibuprofen is also taken for many other types of pain, but the prescription doses > 400 mg included here are reserved for musculoskeletal indications. After ibuprofen, diclofenac was prescribed second most often, followed by etoricoxib.

Among the nonopioid analgesics, the frequent prescription of metamizole is striking. In RA, every third person was prescribed metamizole, regardless of whether seropositive RA was coded or not. However, the proportion of long-term prescriptions of metamizole is considerably lower at 6–9%. Metamizole is recommended as an alternative to NSAIDs or paracetamol, especially in older people, because of its good analgesic efficacy and rather low potential for interaction with concomitant diseases or drugs [10]. In fact, in the >70-year-old age group, a decline in NSAIDs with a simultaneous increase in prescriptions of metamizole and opioids was observed. However, metamizole is not indicated for chronic pain because of its short half-life. Due to possible agranulocytosis as well as hypersensitivity reactions and aplastic anaemia following metamizole intake, regular blood count monitoring is required in Germany. In the USA and also in some European countries, metamizole was withdrawn from the market in the 1970s after an accumulation of reports of agranulocytosis, and a prescription requirement was introduced in Germany [11]. For this reason, the prescription prevalence varies greatly between countries. The frequent use of metamizole in Germany is described in other geriatric populations [11, 12]. The small proportion of paracetamol and acetylsalicylic acid only represents the prescribed proportion. Both substances are mainly obtained by the patients directly from the pharmacy, so that no statement can be made about the frequency of intake.

In patients with RA, the use of opioids has increased significantly in the USA during the last 15 years [13, 14]. According to data from the USA, rheumatologists prescribed opioids to up to 40% of their RA patients [14, 15]. In a multicentre RA cohort from Columbia, even more than half of the patients were prescribed opioids, many of them with a treatment duration of more than 12 months and despite a DMARD and glucocorticoid therapy [16]. There are also data from the UK, France and Switzerland on increasing rates of opioid prescribing in patients with chronic pain [17–19]. In the COPERS study from the UK, 59% of the patients with musculoskeletal pain were prescribed opioids and 40% had more than three prescriptions of strong opioids within a year [20]. In the US PSOAS cohort, the frequency of opioid prescriptions was 22% (short-term) and 10% (long-term) for patient with ankylosing spondylitis (AS) [21]. In a systematic review from Germany that included all publications until 2016, an increase in opioid prescriptions with a clustering of strong opioids, especially fentanyl, in noncancer patients was observed [22]. With regard to long-term prescriptions, an earlier analysis of the BARMER GEK data showed that 1.3% of the insured persons with nontumour-related pain received opioids for at least three consecutive quarters of a year [9]. However, these latter studies do not specifically refer to patients with rheumatic diseases. The data from the present study show that the proportion of insured persons with a diagnosis of RA, axSpA, PsA or SLE and at least one opioid prescription in a year, ranging from 17–21%, is lower than reported prescription rates from other countries, and has not increased overall over the years. However, strong opioids are prescribed more frequently than in the past and the proportion of long-term prescriptions has increased considerably. The frequent long-term prescription of opioids contrasts with the low evidence for its benefit in relation to the high number of side effects. International and German recommendations all have in common that there is no evidence for a long-term use of opioids; therefore the overall recommendation is to use opioids only in exceptional cases and in the short term, whereby NSAIDs are generally preferable as evidence-based first-line therapy [23, 24]. The indication for opioid use should be reconsidered for any patient with a long-term prescription. Both the specifics of pain management in older patients [10] and the rheumatologic recommendations for the use of NSAIDs (nonsteroidal anti-inflammatory drugs) [25] and coxibs [26] should be taken into account.

Opioid therapy is only recommended in exceptional cases and for short term use

As the analgesic prescriptions cannot be assigned to their indication, they may also have been prescribed due to comorbidities. In the available data, there are relevant indications such as osteoarthritis, osteoporosis and also malignancy diagnoses. Since three out of four prescriptions were issued by general practitioners, no conclusion can be drawn from this to the indication. It is encouraging that rheumatologists hardly prescribe opioids. In the case of opioid therapy, the rheumatologist should be consulted not only regarding the indication, but also due to adverse drug effects and possible drug interferences with the existing anti-inflammatory therapy. In the NDB, opioid prescriptions were documented in 4% (SLE) to 10% (axSpA) of rheumatology patients in 2018.

In the PROCLAIR study, it was already investigated which patients are prescribed analgesics particularly frequently. Among the insured persons with a diagnosis of RA, middle-aged persons (50–59 years), women, insured persons with a positive rheumatoid factor and patients in rheumatology care received NSAIDs slightly more frequently; however, the proportion was also above 45% in the other patient groups [2]. A total of 2500 insured persons had provided further information in a written survey. In this sample, 2 out of 3 patients received analgesics, with frequencies varying from 46% (no to little pain) to 82% (severe pain) [27]. Again, metamizole was the most commonly prescribed analgesic (24%), followed by ibuprofen, diclofenac and tilidine/naloxone. In patients with more severe pain, fewer NSAIDs were prescribed, but more WHO step II analgesics (tramadol and tilidine/naloxone). The most common WHO step III drugs were oxycodone (6.4%) and morphine (5.9%) among respondents with severe pain. Among respondents with AS, patients in rheumatology care had received significantly more frequently NSAIDs (69% vs. 51%) and nonopioid analgesics (27% vs. 19%), and a trend towards a more frequent use of opioids (17.6% vs. 14.5%) was observed [28]. The survey also showed a significantly more frequent prescription of all analgesic drugs in the presence of depressive symptoms [29].

In the NDB, which includes data from patients in rheumatology practices and clinics, the documented analgesic use is lower. Opioids are most commonly prescribed to patients reporting severe pain in axSpA. Overall, NSAID prescriptions for RA, AS and PsA have decreased in the 2000s coinciding with the increase in biologic therapies within the NDB [30]. In contrast, other nonopioid analgesics are prescribed more frequently than before. In an earlier evaluation of the NDB, it had already been noticed that patients with seronegative RA received NSAIDs less frequently, but analgesics and opioids more frequently [1]. In the present study, these differences were marginal regarding M05 (seropositive RA) and M06 (seronegative or other RA) diagnosis, although the groups differed significantly in the frequency of DMARD prescriptions.

### Possibilities for the reduction of analgesics

In the PROCLAIR study, prescriptions of NSAIDs (85% to 60%), analgesics (33% to 23%) and opioids (25% to 20%) decreased in the year following the initiation of tumour necrosis factor inhibitor therapy in patients with axSpA [31]. Data from registries in Finland also showed that in children with juvenile idiopathic arthritis, the initiation of DMARD therapy (predominantly methotrexate) significantly reduced the use of NSAIDs, paracetamol and opioids [32]. Both studies demonstrate the benefit of an effective anti-inflammatory therapy for pain reduction and thus the possibility to reduce analgesic prescriptions.

### Limitations and strengths

Claims data only contain prescribed medications. Particularly in the area of analgesics, a large number of substances is available over the counter and these are not recorded in this evaluation but only the proportion of prescription dosages (ibuprofen). Furthermore, claims data do not contain clinically validated diagnoses and the prescriptions are not assigned to the diagnoses, i.e. it is not clear from the data whether the analgesic was prescribed in the context of the rheumatic disease or due to other causes of pain. In contrast to the NDB, claims data represent nationwide nonselected prescription rates, which also include all outpatient prescriptions of other physician groups.

## Conclusions

- Even though the prescription of opioids among insured persons with inflammatory rheumatic diagnoses is not as high as in other countries, we have nevertheless observed a significant increase in long-term prescriptions and in strong opioids over time, although a broader spectrum of effective DMARDs (disease-modifying antirheumatic drugs) or biologics is available today.
- Fortunately, the “opioid epidemic”, which has led to considerable problems in the USA, among others, is not observed to the same extent for rheumatologic patients in Germany. Overall, it was seen that rheumatologists are very restrictive with the prescription of opioids.

## Declarations

For this article no studies with human participants or animals were performed by any of the authors. All studies performed were in accordance with the ethical standards indicated in each case.

The supplement containing this article is not sponsored by industry.
